# 2477. Characteristics of patients infected with Carbapenem-resistant *Acinetobacter baumannii* complex and Carbapenem-resistant Enterobacterales in Tennessee, 2016–2021

**DOI:** 10.1093/ofid/ofad500.2095

**Published:** 2023-11-27

**Authors:** Dipen Patel, Glodi Mutamba, Raquel Villegas, Ashley Gambrell, Cherlly Bailey, Jacquelyn Mounsey, Daniel Muleta

**Affiliations:** HAI/AR, Tennessee Department of Health, Nashville, Tennessee; Tennessee Department of Health, Nashville, Tennessee; State of Tennessee, Nashville, Tennessee; Tennessee Department of Health, Nashville, Tennessee; Tennessee Department of Health, Nashville, Tennessee; TN EIP, Nashville, Tennessee; Tennessee Department of Health, Nashville TN, Antioch, Tennessee

## Abstract

**Background:**

The spread of Carbapenem-resistant pathogens is a growing global public health concern. Tennessee conducts surveillance of Carbapenem-resistant Enterobacterales (CRE) since 2011 and participates in Multi-site Gram-negative Surveillance Initiative (MuGSI) in Davidson County and seven surrounding counties since 2014.

**Methods:**

We analyzed the CRE and Carbapenem-resistant *Acinetobacter baumannii* complex (CRAB) incident cases reported to the MuGSI project from 2016–2021. A CRE case is defined as the isolation of *Escherichia coli, Enterobacter* species and *Klebsiella* species from normally sterile site or urine, and resistant to ≥1 carbapenem. A CRAB case is defined as the isolation of *A.baumannii* complex from a normally sterile site or urine (2016-2020). In 2021, cultures from a lower respiratory tract or wound were added. Incident case is defined as a report of the first case in 30 days. Data analysis was performed using SAS Version 9.4.

**Results:**

One hundred nine CRAB and 467 CRE cases were reported from 2016–2021. The proportion of males was higher for CRAB (62.39% vs 37.61%, p< 0.0001), while the proportion of females were higher for CRE cases (68.74% vs 31.26%, p< 0.0001). The mean age was 65 and 60 years for CRE and CRAB case respectively. The incidence rate of CRAB was significantly higher in non-white populations (15.90 per 100,000) compared to white populations (4.86 per 100,000) (p< 0.0001). Smoking was more prevalent among CRAB patients (27.52%) than CRE patients (16.49%) (p=0.0076). The prevalence of neurological health conditions was higher in CRAB than CRE (59.63% vs 38.33%; p< 0.0001), as were renal conditions (38.53% vs 26.12%; p=0.0097) and diabetes (48.62% vs 37.69%; p=0.0357). The prevalence of urinary tract infections was higher in CRE than CRAB cases (52.29% vs 77.09%; p< 0.0001). CRAB (95%) and CRE (65%) cases were healthcare-associated infections.

**Conclusion:**

Patients with CRAB had significantly higher prevalence of neurological, renal, diabetes and smoking history, while UTI was more common in patients with CRE. CRAB incidence was significantly higher in non-whites than whites. More research is needed to explain the disparities and reduce infection burden in vulnerable populations.

**Disclosures:**

**All Authors**: No reported disclosures

